# Incidence and risk factors contributing to ileus after posterior approach for lumbar surgery: a retrospective study

**DOI:** 10.3389/fsurg.2025.1528409

**Published:** 2025-09-19

**Authors:** Rui Bao, GuoLei Liang, YiNan Liu, Dan Wang, Rui Ma, YiZhi Cui, YangYang Tian, Le Wang, Fulin Guan

**Affiliations:** ^1^ Acupuncture and Moxibustion Department 1, Third Affiliated Hospital of Heilongjiang University of Chinese Medicine, Harbin, Heilongjiang, China; ^2^ Orthopedic Ward 4, First Affiliated Hospital of Harbin Medical University, Harbin, China; ^3^ Neurology Department 1, Beijing University of Chinese Medicine, Beijing, China; ^4^ Second Affiliated Hospital, Heilongjiang University of Chinese Medicine, Harbin, Heilongjiang, China; ^5^ Dongfang Hospital, Beijing University of Chinese Medicine, Beijing, China

**Keywords:** risk factor (RF), postoperative ileus (POI), posterior lumbar surgery, retrospective study, lumbar surgery

## Abstract

**Objective:**

This retrospective study aimed to identify the incidence and possible predictive factors associated with ileus after posterior approach for lumbar surgery.

**Patients and methods:**

A total of 267 patients who underwent a posterior approach for lumbar surgery between 2012 and 2020 were analyzed in this study. The differences between the two groups and the risk factors of ileus were explored.

**Results:**

Patients’ characteristics showed no significant differences between the two groups. This revealed that gender, age, smoking, hypertension, and diabetes were not associated with postoperative ileus (POI). Patients with POI would increase length of hospital stay significantly (*p* = 0.015). Operative segment [odds ratio (OR): 1.40, 3.33; *p* = 0.04 and 0.02], postoperative blood potassium (OR: 0.92, 0.31; *p* = 0.04), and previous abdominal surgery (OR: 3.02, *p* = 0.01) were significant independent risk factors for POI. Operation time, blood loss, and anesthesia type were not considered risk factors for POI.

**Conclusion:**

Postoperative ileus can increase the length of hospital stay significantly. Operative segment, postoperative blood potassium, and previous abdominal surgery were significantly associated with POI, which should be highlighted in the preoperative evaluation.

## Introduction

Postoperative ileus (POI) is a complication lasting more than 3 days postoperatively. It involves abnormal gastric motility and typically results in bloating, abdominal distension, constipation, and nausea (1). Treatment of POI includes conservative management, such as nasogastric intubation, electrical stimulation, and pharmacologic agents, such as nil per os. The *μ*-opioid receptor antagonist can be used to reduce POI (2). In addition, researchers claim that preventing medication therapy is not effective in reducing POI (3). Surgery is rarely used to manage POI and is reserved for failure of pharmacological treatment (4–6). POI affects approximately 3.5% of patients undergoing spinal surgery (7). POI is a known complication after anterior lumbar interbody fusion (ALIF) due to the anterior abdominal approach to the vertebral column. It is frequently associated with prolonged length of hospital stay (LOS) and increased healthcare costs (8, 9). Some research claims that many spinal surgeons prefer an “access surgeon” to perform the exposure, believing it may be helpful to reduce the rate of POI (8). Compared with posterior lumbar interbody fusion (PLIF), ALIF has a higher incidence of POI (74.9 vs. 26.0 per 1,000) (7). For this reason, most studies have focused on investigating ileus in patients who underwent ALIF or lateral lumbar interbody fusion (LLIF). The incidence after posterior lumbar surgery remains unclear, and the literature on POI after posterior lumbar surgery is scarce (10). Even though the incidence of POI in PLIF is generally lower than in ALIF, it remains a postoperative complication that can increase length of hospital stay and place a burden on the healthcare system. Identifying potential POI risk factors may enhance surgeon awareness and support perioperative decision-making. At the same time, targeted preventive measures for patients at higher risk could help reduce the incidence of POI, thereby decreasing medical burden and costs (11). These results highlight the need for ongoing evaluation of both the incidence of POI and the risk factors associated with its occurrence in patients undergoing posterior lumbar surgery.

## Materials and methods

The project was conducted between January 2012 and December 2020 and included 300 consecutive posterior lumbar spine surgical procedures. Data collection was not planned before the operations. All surgeries were performed by two surgeons and involved either lumbar decompression or PLIF. Surgical indications included lumbar disc herniation, lumbar instability, lumbar stenosis, or lumbar spondylolisthesis. For each surgery, patient age, sex, tobacco use, history of prior lumbar spine or abdominal surgery, surgery category (lumbar decompression vs. PLIF), number of operative levels, estimated blood loss, surgery duration, type of anesthesia, and presence of diabetes mellitus or hypertension were recorded. Indications for lumbar decompression included symptoms of spinal nerve compression such as pain, altered sensation, muscle weakness or dysfunction, and gait abnormalities caused by degenerative spinal stenosis and/or disc herniation. The type of anesthesia, determined by the anesthetist, was either combined spinal and epidural anesthesia or general anesthesia.

Prolonged POI was defined as the presence of abdominal distension, nausea, vomiting, and delayed passage of gas and stool persisting beyond the fourth postoperative day. Patients suspected of having POI based on physical examination underwent abdominal X-ray to confirm the diagnosis. Both prolonged and recurrent POI were taken into consideration.

This study was reviewed and approved by Institutional Review Board (IRB).

Demographic characteristics and clinical data were compared between the POI and normal groups using quantitative variables and the *χ*^2^ test for categorical variables. Univariate analysis was performed to estimate the unadjusted odds ratio (OR) and 95% confidence interval (CI). Logistic regression analysis was conducted, with two-sided *p*-values <0.05 considered statistically significant. All statistical analyses were performed using SAS version 9.2 (SAS Institute, Cary, NC, USA).

## Results

### Patient characteristics

In total, 299 patients were enrolled who underwent primary lumbar discectomy or PLIF by two senior surgeons in our department. This 4-year follow-up was completed in September 2021, with a follow-up rate of 89.0% (267/300). A total of 24 patients could not be contacted, and nine patients died due to unrelated causes over the 10 years. Of the 267 patients, 18 (6.74%) developed ileus after lumbar spine surgery. All cases were diagnosed by abdominal X-ray and physical examination. The mean age of participants was 45.7 years (range 19–91 years; SD = 14.6 years). The cohort consisted of 135 (50.2%) men and 137 (49.8%) women. The average body mass index was 30.2 kg/m^2^ (range 16–55 kg/m^2^; SD = 6.3 kg/m^2^). In total, 89 patients underwent posterior lumbar decompression, and 178 (66.6%) patients underwent lumbar fusion. The number of operative levels was one (69.3%; *n* = 185), two (22.5%; *n* = 60), and more than two (7.1%; *n* = 19). Of the 18 ileus cases, eight had a LOS less than 5 days and 10 had a LOS greater than 5 days. Among patients without ileus, 182 had a LOS less than 6 days and 67 patients had a LOS greater than 5 days (*p* = 0.015). Table 1 shows that patient characteristics such as gender, age, smoking, hypertension, and diabetes were not associated with POI.

**Table 1:** Characteristics of two groups of patients in this series.

Characteristics		POI (%) (*n* = 18)	Normal (%) (*n* = 249)	*P*
Gender	Male	12 (66.67)	122 (49.00)	0.15
	Female	6 (33.33)	127 (51.00)	
Age (year)	≤40	2 (11.11)	54 (21.69)	0.40
	41–50	5 (27.78)	67 (26.91)	
	51–60	7 (38.89)	62 (24.90)	
	≥60	4 (22.22)	66 (26.51)	
Hospital stay (days)	≤5	8 (44.44)	182 (73.09)	0.015
	>5	10 (55.56)	67 (26.91)	
Smoking	No	11 (61.11)	177 (71.08)	0.37
	Yes	7 (38.89)	72 (28.92)	
Alcohol abuse^a^	No	13 (72.22)	181 (73.49)	1.00
	Yes	5 (27.78)	65 (26.51)	
Hypertension	No	14 (77.78)	186 (85.28)	1.00
	Yes	4 (22.22)	63 (14.72)	
Diabetes	No	15 (83.33)	203 (81.53)	1.00
	Yes	3 (16.67)	46 (18.47)	
ECG^b^	Normal	11 (61.11)	137 (58.55)	0.83
	Abnormal	7 (38.89)	97 (44.45)	

^a^ Three samples missing.

^b^ Fifteen samples missing.

### Univariate analysis and multivariate analysis

Table 2 presents the results of the univariate analysis of demographic and surgical risk factors for POI. Most patients in this study underwent general anesthesia, including 72.22% in the POI group and 65.06% in the normal group, with no significant differences between the two groups. The majority of patients underwent posterior decompression and fusion: 61.11% in the POI group and 56.63% in the normal group. Decompression alone was performed in 11.11% of POI patients and 9.64% of normal patients. Regarding the number of operative levels, 66.67% of POI patients and 69.88% of normal patients had one level operated on, 16.67% and 23.69% had two levels, and 16.67% and 6.43% had more than two levels, respectively. Most patients had no prior surgical history, with 66.67% in the POI group and 79.12% in the normal group. Univariate analysis showed no significant differences for these factors.

**Table 2:** Univariate analysis of risk factors for POI after posterior lumbar surgery.

Characteristics	POI no. (%)	Normal no. (%)	OR (95% CI)	*P*
Anesthesia type				
General	13 (72.22)	162 (65.06)	1.00	
Spinal epidural	5 (27.78)	87 (34.94)	0.72 (0.25–2.08)	0.54
Surgical type				
PLIF	5 (27.78)	84 (33.73)	1.00	
Decompression and fusion	11 (61.11)	141 (56.63)	1.31 (0.44–3.90)	0.62
Decompression	2 (11.11)	24 (9.64)	1.40 (0.26–7.67)	0.70
Surgical level^a^				
1	12 (66.67)	173 (69.88)	1.00	
2	3 (16.67)	57 (23.69)	0.76 (0.21–2.78)	1.00
More than 2	3 (16.67)	16 (6.43)	2.70 (0.69–10.58)	0.15
Operation time (h)^b^				
≤2	6 (33.33)	97 (40.08)	1.00	
2–3	8 (44.44)	84 (34.71)	1.54 (0.51–4.61)	0.44
≥3	4 (22.22)	61 (25.21)	1.06 (0.29–3.91)	1.00
Blood loss (ml)^c^				
≤100	10 (55.56)	96 (41.20)	1.00	
100–300	5 (27.78)	86 (36.91)	0.56 (0.18–1.70)	0.30
≥300	3 (16.67)	51 (21.89)	0.57 (0.15–2.14)	0.55
Postoperative blood potassium^d^				
≤3.60	9 (50.00)	80 (33.90)	1.00	
3.60–4.00	4 (22.22)	97 (41.10)	0.37 (0.11–1.24)	0.09
≥4.00	5 (27.78)	59 (25.00)	0.75 (0.24–2.36)	0.63
Previous abdominal surgery				
No	12 (66.67)	197 (79.12)	1.00	
Yes	6 (33.33)	52 (20.88)	1.89 (0.68–5.29)	0.24

^a^ Three samples missing.

^b^ Seven samples missing.

^c^ Sixteen samples missing.

^d^ Thirteen samples missing.

Table 3 presents the results of the multivariate analysis of demographic and surgical risk factors for POI. Operative segment (OR: 1.40, 3.33; *p* = 0.04 and 0.02), postoperative blood potassium (OR: 0.92, 0.31; *p* = 0.04), and previous abdominal surgery (OR: 3.02; *p* = 0.01) were identified as significant independent risk factors for POI. Operation time, blood loss, and anesthesia type were not considered risk factors for POI.

**Table 3:** Multivariate analysis of risk factors for POI after posterior lumbar surgery.

Characteristics	*B*	S.E.	Wald	OR (95% CI)	*P*
Anesthesia type	0.21	0.47	0.18	1.03 (0.32–2.06)	0.99
Decompression and fusion	0.65	0.53	1.48	1.91 (0.67–5.39)	0.22
Decompression	0.34	0.87	0.15	1.40 (0.26–7.67)	0.70
Two surgical levels	0.34	0.51	0.43	1.40 (0.51–3.86)	0.04
More than 2 levels	1.20	0.63	3.66	3.33 (0.97–11.41)	0.02
Operation time 2–3 h	−0.37	0.50	0.55	1.44 (0.55–3.83)	0.46
More than 3 h	−0.06	0.59	0.02	0.99 (0.31–3.18)	0.98
Blood loss 100–300 ml	−0.34	0.51	1.46	0.71 (0.26–1.91)	0.47
Blood loss ≥300 ml	−0.16	0.57	0.76	0.86 (0.28–2.60)	0.74
Postoperative blood potassium 3.60–4.00	−0.70	0.54	1.71	0.92 (0.23–1.03)	0.04
Postoperative blood potassium ≥4.00	−0.13	0.69	0.03	0.31 (0.12–0.98)	0.03
Previous abdominal surgery	0.70	0.47	2.29	3.02 (1.73–12.02)	0.01

## Discussion

Postoperative paralytic ileus (POI) after lumbar surgery can significantly increase the length of stay and cost of care. It is characterized by slow or absent gastrointestinal motility secondary to surgery, primarily caused by neural reflexes, inflammation, and neurohumoral peptides (12). The small intestine typically regains motility within hours after surgery, while the stomach and colon recover later (13). If symptoms persist beyond this period, it can be considered abnormal. In our study, the incidence of POI after posterior lumbar surgery was 6.74%, which is higher than the reported incidence for ALIF and LLIF, ranging from 7.4% to 7.0% (7, 10).

Previous retrospective studies have shown that the risk of developing ileus is significantly higher after anterior lumbar spinal fusion (7.5%) compared to posterior lumbar spinal fusion (2.6%), with the highest risk (8.4%) occurring when both anterior and posterior approaches are used during spine surgery (7). In our study, the incidence of ileus after PLIF is 6.74%, which is lower than previously reported. The difference may be attributable variations in ethnicity, sample size, and, most importantly, surgical approach. The anterior approach increases the risk of POI because the abdominal organs, such as the intestines, kidneys, and blood vessels, are displaced when accessing the retroperitoneal space.

Patients who developed ileus typically experience longer LOS in hospital and incur greater hospital costs (9, 14). Although our LOS cutoff was longer than average, we still found that POI prolonged the length of hospital stay. Our results expand on the conclusion by showing that POI increases the length of stay not only after anterior approaches but also after posterior lumbar surgery.

Reported incidence of ileus after lateral lumbar surgery is approximately 7%, which is higher than the incidence we observed with posterior approaches. In our study, operative segment, postoperative blood potassium, and previous abdominal surgery were identified as risk factors for POI, whereas operating time and blood loss were not. This finding is consistent with previous research (10).

Previous research revealed that male gender was a risk factor for POI, which is different from our results, possibly due to differences in race and sample size. Multi-level operations were also found to increase POI risk, consistent with a previous study investigating ALIF (15, 16). Prior ALIF research has also shown that increased intraoperative blood loss is a risk factor for prolonged POI, a finding different from our own, likely due to the differences in surgical approach (15). Although general anesthesia can contribute to postoperative intestinal dysmotility, research indicates that longer anesthesia duration does not necessarily prolong ileus (17). In our study, no difference was observed between anesthesia types.

The present study has some limitations, such as its retrospective nature. Postoperative therapies may influence the occurrence of ileus; however, this information was not available in our dataset, and future studies focusing on this factor may be helpful. We were unable to obtain information regarding the specific anesthetic drugs used, which could be helpful to POI risk. Finally, physicians may not always document ileus as a complication, especially if it presents mildly and is perceived as a normal physiological response to surgery, even when its duration is longer than expected (18).

## Conclusion

Based on a 9-year follow-up, the incidence of POI after posterior lumbar surgery was 6.64%. POI significantly increased the length of hospital stay. Statistical analysis identified operative segment, postoperative blood potassium, and previous abdominal surgery as risk factors for POI.

## Data Availability

The raw data supporting the conclusions of this article will be made available by the authors, without undue reservation.
